# Supplementary material for: Comparison of participant-collected nasal and staff-collected oropharyngeal specimens for human ribonuclease P detection with RT-PCR during a community-based study
Source: PLoS One. 2020 Oct 7;15(10):e0239000. doi: 10.1371/journal.pone.0239000 (PMC7540885; doi:10.1371/journal.pone.0239000)
Supplement: S1 File — The staff member fills out this questionnaire at the home visit with the help of the ORCHARDS participant and parent(s). (PDF) [file pone.0239000.s001.pdf]

OMB Approved
Control # 0920-1039

U.S. Department of Health and Human Services (HHS)
Centers for Disease Control and Prevention (CDC)

ORegon CHild Absenteeism due to Respiratory Disease Study

Acute Respiratory Infection and Influenza Surveillance Form

School ID: 4K P N B R M H

Participant ID: _____________

Age: __________________

Date of Collection: ______________

Criteria for patient selection and testing

- symptom onset within 4 days
- any two of the following
  - rhinorrhea
  - nasal congestion
  - sneezing
  - sore throat
  - cough
  - fever

- -

Days between illness onset and today's visit: ____ days

Exposure to a similar illness 1-3 days prior to ARI onset? Yes No

Likely Source: Classmate Friend Family Member (Adult / Sibling) Other: ___________________

Household Member (circle if living in household, check box if ill with similar ARI):

Grandmother ☐	Grandfather ☐	Mother (*Female Guardian)* ☐	Father (*Male Guardian)* ☐
O/Y Sibling #1 ☐	O/Y Sibling #2 ☐	O/Y Sibling #3 ☐	Other Adult:_________ ☐
O/Y Sibling #4 ☐	O/Y Sibling #5 ☐	O/Y Sibling #6 ☐	Other Child:_________ ☐

Recent Travel? Yes No **Recent Exposure to Farm Animals?** Yes No

Severity of Illness (circle)**:** Mild Moderate Severe

Race: White Am Indian or Ak Native Asian Black Native Hawaiian or Other Pacific Islander

Ethnicity: Hispanic Non-Hispanic

Measured Temperature ___ ^{o}F **Antipyretic use within the last 6 hours?** Yes No

Symptoms (circle all that are present)**:**

Fever	Chills	Cough	Wheezing	Runny Nose	Sore Throat
Malaise	Myalgia	Arthralgia	Nasal Congestion	Headache	Ear Pain
Anorexia	Vomiting	Abdominal Pain	Diarrhea	Conjunctivitis	Other: ____________

Influenza antiviral treatment for this illness prior to this visit? Yes No

Seasonal influenza vaccine prior to this illness? Yes No

Visit to health care provider for this illness prior to home visit? Yes (specify when __________________) No

Visit to health care provider planned in next few days? Yes (specify when __________________) No

Indicate Specimen Type(s) for PCR testing: nasopharynx posterior pharynx

- - - - - - - - - - **ITEMS BELOW THIS LINE FOR LABORATORY ONLY** - - -

Sofia Result: ☐ **flu A** ☐ **flu B** ☐ **both** ☐ **negative** ☐ **invalid**

Sample Code: _ORCHARDS_ - _____ - ____ - ______
site week staff sample

____

Public reporting burden of this collection of information is estimated to average 30 minutes per response, including the time for reviewing instructions and completing and reviewing the collection of information. An agency may not conduct or sponsor this survey, and a person is not required to respond to a collection of information, unless it displays a currently valid OMB Control Number. Send comments regarding this burden estimate or any other aspect of this collection of information, including suggestions for reducing this burden to CDC/ATSDR Reports Clearance Officer, 1600 Clifton Road NE, MS D-74, Atlanta, Georgia 30333; ATTN: PRA 0920-1039.
