# Supplementary material for: Comparison of participant-collected nasal and staff-collected oropharyngeal specimens for human ribonuclease P detection with RT-PCR during a community-based study
Source: PLoS One. 2020 Oct 7;15(10):e0239000. doi: 10.1371/journal.pone.0239000 (PMC7540885; doi:10.1371/journal.pone.0239000)
Supplement: S2 File — Each Household Substudy participant receives this questionnaire in the Household Substudy Kit and fills it out on Day 0 and Day 7 by him/herself or with the help of a parent. (PDF) [file pone.0239000.s002.pdf]

ORCHARDS HOUSEHOLD STUDY FORM

School ID: ______________

Participant ID: ___________

NASAL SWAB

ID

HOUSEHOLD MEMBER NAME: ____________________

RELATIONSHIP TO STUDENT: _____________________

Age: _____ **Do you work outside the home?** Yes No **Number of bedrooms:** _____

Gender: F M **Do you attend school?** Yes No **Do you attend Daycare?** Yes No

- -

TODAY Day 0 (___ / ___ / ___)

-Did you receive an influenza vaccine this year (after August 1, 2019)? Yes No

Have you had cold or flu-like symptoms in the past 7 days? Yes No (if **No**, then you are done until next week)

If yes: How many days ago did your symptoms start? _______

How severe are/were your symptoms? Mild Moderate Severe

What symptoms have you had in the past 7 days? (circle all that have been present)

Fever Chills Cough Runny Nose Sore Throat

Tiredness Body Aches Headache Poor Appetite Nasal Congestion

Were you seen by a healthcare provider? Yes No **Where?** Usual Clinic Urgent Care ER

What diagnosis were your given? ____________________

Were you given an antibiotic or antiviral medication? Yes No ______________________

Were you sent to the hospital? Yes No

Did you miss school or Work? Yes No **If yes, how many days did you miss?** ______

- -

FOLLOW-UP Day 7 (___ / ___ / ___)

Have you had cold or flu-like symptoms in the past 7 days (since our previous visit)? Yes No

If yes: How many days ago did your symptoms start? _______

How severe are/were your symptoms? Mild Moderate Severe

What symptoms have you had in the past 7 days? (circle all that have been present)

Fever Chills Cough Runny Nose Sore Throat

Tiredness Body Aches Headache Poor Appetite Nasal Congestion

Were you seen by a healthcare provider? Yes No **Where?** Usual Clinic Urgent Care ER

What diagnosis were your given? ____________________

Were you given an antibiotic or antiviral medication? Yes No ______________________

Were you sent to the hospital? Yes No

Did you miss school or Work? Yes No **If yes, how many days did you miss?** ______
