# Supplementary material for: Comparison of participant-collected nasal and staff-collected oropharyngeal specimens for human ribonuclease P detection with RT-PCR during a community-based study
Source: PLoS One. 2020 Oct 7;15(10):e0239000. doi: 10.1371/journal.pone.0239000 (PMC7540885; doi:10.1371/journal.pone.0239000)
Supplement: S3 File — Each Household Study Kit came with instructions for collection that participants could voluntarily use for guidance. (PDF) [file pone.0239000.s003.pdf]

ORCHARDS - FAMILY
Oregon CHild Absenteeism due to Respiratory Disease Study

▶ Day 0

1. Each family member should have their own bag with an ORCHARDS Household Study Form inside.
    - The sheet is split into two portions: “TODAY” (Day 0) and “FOLLOW-UP” (Day 7)
    - Please fill out the top portion of the sheet (“TODAY”, Day 0) for each family member
    - TIP: Answering “no” to flu symptom question means the top portion of that form can remain blank
2. Collect a nasal swab specimen for each household member (excluding the child who participated in ORCHARDS).
    - Insert swab labeled with a GREEN dot into one nostril and rotate swab **3 times** against the nasal wall
    - Place the swab into the test tube of viral transport media labeled with a GREEN dot, snap off the excess swab shaft, & screw the cap on tightly
    - Place test tube back in small biohazard bag, and small biohazard bag back in the corresponding family member’s bag
3. Place all family members’ bags back into the large family bag, and place in the refrigerator (preferably in the middle of the fridge, to prevent freezing).

(Turn over for Day 7 directions!)

▶ Day 7

1. Today will be a repeat of Day 0.
    - Please fill out the bottom portion of each family member’s ORCHARDS Household Study Form (“FOLLOW-UP”, Day 7).
    - REMINDER: Child who participated in ORCHARDS will participate today.
2. Collect a 2nd nasal swab specimen for each household member (including the child who participated in ORCHARDS), following the same instructions as Day 0.
    - Use swab labeled with a RED dot
    - Place the swab into the test tube of viral transport media labeled with a RED dot, snap off the excess swab shaft, & screw the cap on tightly
    - Place test tube back in small biohazard bag, and small biohazard bag back in the corresponding family member’s bag
3. Place all family members’ bags back into the large family bag, and place in the refrigerator until ORCHARDS staff comes to collect.
    - Please make sure you have signed all consent forms!

If you have questions at any point, please call the ORCHARDS study phone number: [redacted]
